# WT1-Related Nephropathy in a Phenotypically Female Child: A Case of Clinical and Genetic Discordance

**DOI:** 10.3390/children12050595

**Published:** 2025-05-02

**Authors:** Mariana Costin, Eliza Elena Cinteză, Anca Croitoru, Ionela-Loredana Popa, Alexandra Stanciu, Irina Popescu, Nicoleta Petre, Bettyna Olivotto, Andrei Căpitănescu, Sofia Resceanu, Elena Cotfasa, Cristina Bologa

**Affiliations:** 1 “Carol Davila” University of Medicine and Pharmacy, 050474 Bucharest, Romania; mariana.costin@umfcd.ro (M.C.); eliza.cinteza@umcfd.ro (E.E.C.); loredana.popa@umfcd.ro (I.-L.P.); 2 Department of Pediatric Nephrology, “M.S. Curie” Emergency Clinical Hospital for Children, 077120 Bucharest, Romania; stanciualexandra5@gmail.com (A.S.); resceanusofia@yahoo.com (S.R.); cozmaelena16@yahoo.com (E.C.); crissoim@yahoo.com (C.B.); 3 Department of Pediatric Cardiology, “M.S. Curie” Emergency Clinical Hospital for Children, 077120 Bucharest, Romania; 4 Department of Pediatric Radiology, “M.S. Curie” Emergency Clinical Hospital for Children, 077120 Bucharest, Romania; irinarosoiu@yahoo.com; 5 Clinical Hospital of Nephrology “Dr. Carol Davila”, 010731 Bucharest, Romania; nicoleta.petre@gmail.com; 6 Department of Dialysis, “M.S. Curie” Emergency Clinical Hospital for Children, 077120 Bucharest, Romania; bettynaolivotto@gmail.com (B.O.); andreicapitanescu@gmail.com (A.C.)

**Keywords:** Frasier syndrome (FS), Denys–Drash syndrome (DDS), end-stage kidney disease (ESKD), thrombotic microangiopathy (TMA), focal segmental glomerulosclerosis (FSGS), Wilms Tumor 1 (WT1)

## Abstract

WT1-related disorders comprise a spectrum of conditions resulting from mutations or deletions of the WT1 gene. Alteration in this gene have been associated with many syndromes, including WAGR syndrome, Denys–Drash syndrome (DDS), Frasier syndrome (FS) and Meacham syndrome. We present the case of an 8-year-old phenotypically female child with symptoms of end-stage kidney disease (ESKD), hypertension and anasarca, requiring renal replacement therapy. This case is distinctive due to its unusual onset, the presence of thrombotic microangiopathy (TMA), and the detection of a heterozygous missense mutation in the *WT1* gene (c.1298G>A, p.Cys433Tyr) located in exon 8, in association with a 46 XY karyotype. The kidney biopsy indicated advanced focal segmental glomerulosclerosis (FSGS) with characteristics of TMA, implying a possible alternative diagnosis. In light of the heightened malignancy risk, the patient had preventative laparoscopic gonadectomy, which revealed rudimentary testicular tissues. The identified genotype points toward a diagnosis of DDS. However, the clinical presentation is more consistent with features typically seen in FS. This discrepancy highlights the significant phenotypic and genotypic overlap between the two syndromes. As a result, there is ongoing discussion in the literature about whether DDS and FS should be considered distinct clinical entities or rather variable expressions along a shared disease spectrum.

## 1. Introduction

WT1-related disorders comprise a range of conditions caused by mutations or deletions in the WT1 gene. The tumor suppressor gene located on chromosome 11p15 encodes the Wilms Tumor 1 transcription factor, which plays a role for renal and gonadal development and is associated with carcinogenesis. Alterations in the WT1 gene are linked to a wide array of illnesses, including WAGR syndrome (marked by Wilms tumor, aniridia, genitourinary malformations, and intellectual disability), Denys–Drash syndrome (DDS), Frasier syndrome, and Meacham syndrome [1].

Frasier syndrome and Denys–Drash syndrome are disorders associated with the WT1 gene, sharing characteristics such as male pseudohermaphroditism, progressive nephropathy, and an increased risk of genitourinary malignancies. They are mainly differentiated by their renal disease and associated genetic mutations. Frasier syndrome is generally attributed to a splice-site mutation in intron 9 of the WT1 gene, characterized by a delayed onset of kidney disease and an elevated incidence of gonadoblastoma [2,3]. In contrast, Denys–Drash syndrome develops due to a mutation in exon 8 or 9 and is linked to early-onset nephropathy caused by widespread mesangial sclerosis, frequently advancing swiftly to end-stage renal disease, alongside an increased risk of Wilms tumor development [4,5]. Meacham syndrome, the rarest WT1-associated illness, encompasses genitourinary, cardiac, and pulmonary abnormalities and is often lethal in early life [1]. The lack of established diagnostic criteria and considerable clinical variability complicate an accurate diagnosis, despite the presence of typical symptoms [1,6].

We present a case of an 8-year-old phenotypically female child diagnosed with end-stage kidney disease, hypertension, and anasarca, requiring renal replacement therapy. This case is characterized by its atypical onset, the development of thrombotic microangiopathy (TMA), and the identification of the WT1 mutation (c.1298G>A, p.Cys433Tyr) in exon 8. Genetic analyses, including whole exome sequencing (WES), confirmed the WT1 mutation and a 46 XY karyotype. The kidney biopsy indicated advanced focal segmental glomerulosclerosis (FSGS) with characteristics linked to thrombotic microangiopathy (TMA), warranting early consideration of an alternative diagnosis such as atypical hemolytic uremic syndrome (aHUS), despite negative complement genetic testing. The patient underwent prophylactic laparoscopic gonadectomy due to a heightened risk of gonadal tumors, with histological investigation revealing rudimentary testicular tissues, but no malignancy was identified. 

The late onset of chronic kidney disease, together with the histopathological results from the renal biopsy, supports the diagnosis of Frasier syndrome. However, existing data indicate that a mutation in exon 8 is more frequently linked to Denys–Drash syndrome. The presence of characteristics from both syndromes complicates the diagnosis process in this case [1,4,7,8]. Ongoing research into the molecular processes of WT1-related illnesses may improve our comprehension of disease pathophysiology and facilitate the creation of more effective therapy strategies [8].

## 2. Case Report

An 8-year-old phenotypically female child showed signs of acute enterocolitis and oliguria accompanied by ocular edema. Prior to the current episode, the patient was asymptomatic, with no history of clinical evaluations, laboratory testing, or medical follow-up documented before admission. Furthermore, there is no reported family history of renal disease. She was initially examined by her general practitioner (GP) and laboratory tests indicated hyperkalemia, hypocalcemia, severe azotemia, and metabolic acidosis. She was referred to our hospital for additional assessment and treatment. 

Upon admission, the patient exhibited somnolence, tachypnea, and respiratory distress, with SpO2 levels ranging from 89% to 94%, necessitating oxygen therapy (4–6 L/min via mask). Additionally, the patient presented with tachycardia (179 beats per minute), hypertension (180/100 mmHg), anasarca, bilateral pulmonary rales, and anuria. 

Preliminary laboratory tests revealed severe metabolic acidosis, normocytic normochromic anemia, leukocytosis with neutrophilia, elevated inflammatory markers, increased LDH with normal bilirubin levels, hypoalbuminemia, elevated pancreatic enzymes, and elevated NT-proBNP and troponin levels (Table 1). Peripheral blood smear analysis demonstrated mild erythrocytes anisocytosis with preserved normochromia and rare schistocytes, and platelet morphology was within normal limits, with occasional macrothrombocytes. 

**Figure 1:**
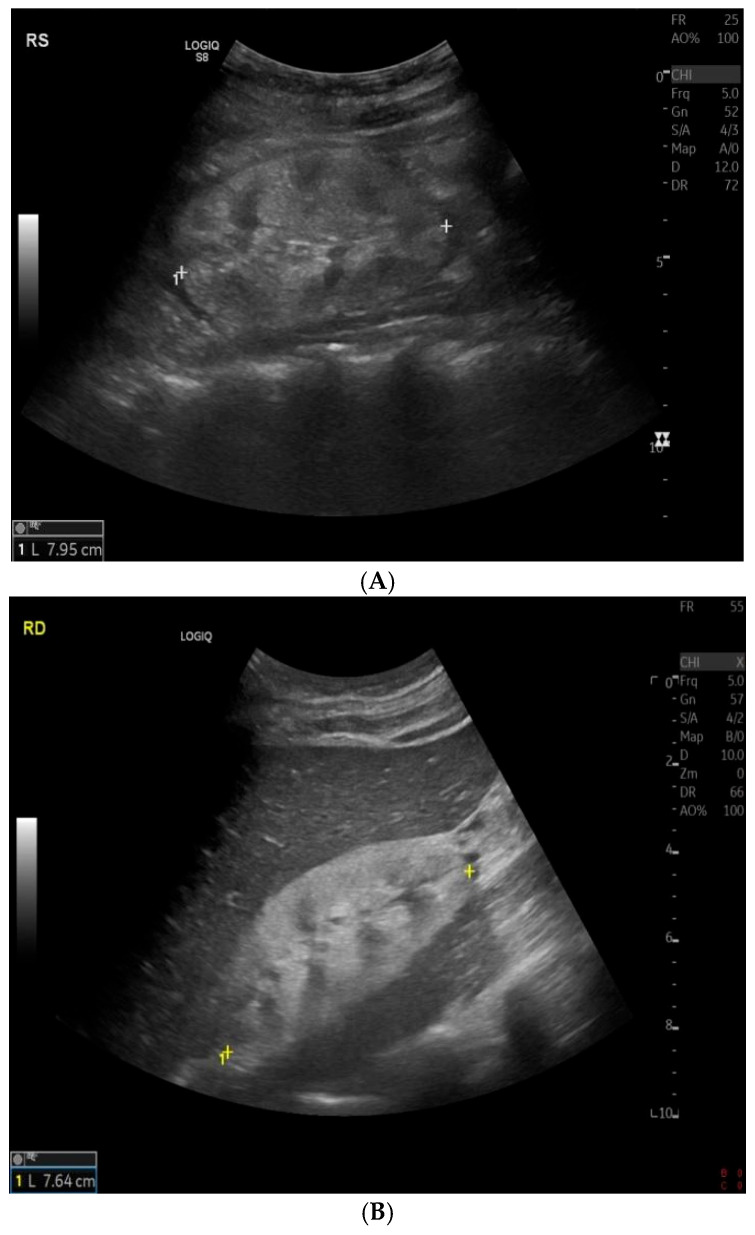
Kidney ultrasound: (**A**) right kidney; (**B**) left kidney—both kidneys have reduced diameters and decreased cortico-medullary differentiation.

The abdomen ultrasonography showed small kidneys with enhanced cortical echogenicity, reduced corticomedullary differentiation, and normal position (Figure 1). 

**Figure 2:**
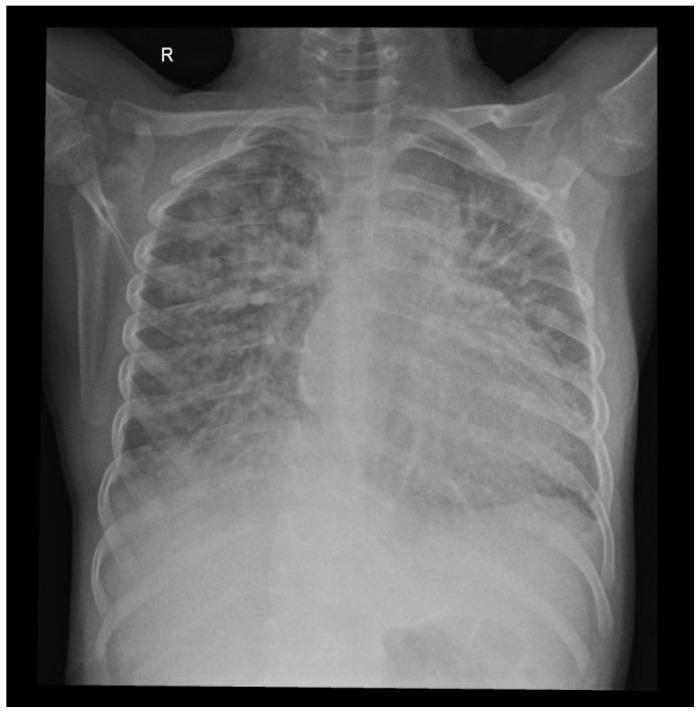
Chest X-ray: pulmonary edema, bilateral diffuse alveolar opacities and cardiomegaly.

The chest X-ray was conducted because of acute respiratory distress upon arrival and showed bilateral diffuse alveolar opacities, characteristic for acute pulmonary edema (Figure 2).

Echocardiography reveals a dilated left ventricle, concentric left ventricular hypertrophy, grade I aortic regurgitation, and grade I mitral regurgitation with left ventricular ejection fraction of 45–50%. The cardiac abnormalities are attributable to the current severe episode; however, the presence of concentric left ventricular hypertrophy raises the question of chronic hypertension and the possibility of an insidious onset of the disease long before diagnosis.

The young child was admitted to the pediatric intensive care unit (PICU), where she underwent intubation and mechanical ventilation for one day due to respiratory distress. She received continuous renal replacement therapy (CRRT) for five days because of severe azotemia and anuria. Intravenous nicardipine was delivered at a starting dose of 1 mcg/kg/minute, with a maximum dose of 4–5 mcg/kg/minute, for hypertension, whereas red blood cell transfusions were provided for severe anemia (Hb = 6 g/dL). Empirical antibiotic therapy with ceftriaxone was commenced; however, all blood, stool, and tracheal aspirate cultures returned negative results.

Subsequent to stabilization, the patient was transported to the nephrology department, where she continued to exhibit oligo-anuria. Vital signs indicated improvement, with blood pressure obtained at 110/76 mmHg, a heart rate of 100 bpm, and SpO2 above 96%. She continued to be dependent on hemodialysis three times a week, and oral antihypertensive treatment was continued.

Due to the patient’s fast advancement to end-stage kidney disease, a differential diagnosis was performed to determine possible underlying causes. Viral screening for HIV, hepatitis B, and hepatitis C returned negative results. The autoimmune panel, which assessed antinuclear antibodies (ANA), anti-double-stranded DNA (anti-dsDNA), ANA immunoblot, and anti-neutrophil cytoplasmatic antibodies (pANCA and cANCA), showed negative results. Following the resumption of diuresis, urinalysis indicated substantial proteinuria (1000 mg/dL) and microscopic hematuria, with urine culture showing no signs of infection. 

**Figure 3:**
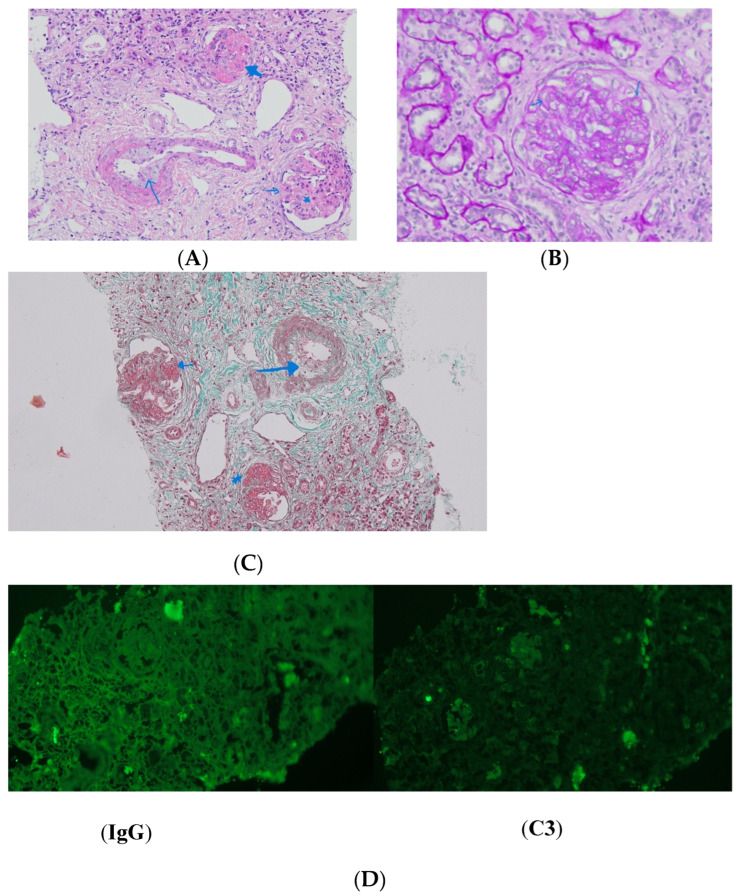
Light microscopy images of the kidney sample: (**A**) Periodic acid Schiff (PAS) staining: the blue arrows highlight a duplicated glomerular basement membrane (GBM). (**B**) Hematoxylin eosin (HE) staining: The thick arrows indicate consolidated glomerulus; the long arrow indicates vascular wall edema; the short arrow indicates segmental sclerosis with capsular adhesion; and the asterisk indicates mesangiolysis. (**C**) Masson’s Trichrome staining: the long arrow indicates vascular wall edema; the short arrow highlights segmental sclerosis and capsular adhesion. The blue asterisk marks glomerulosclerosis. (**D**) Immunofluorescence shows negative IgG and C3 staining in the glomeruli, with positive staining in tubular casts.

The kidney biopsy revealed progressive glomerulosclerosis along with features diagnostic for TMA. A light microscope revealed, on tissue sections stained with hematoxylin-eosin (HE), Periodic acid Schiff (PAS), and Masson’s Trichrome (MT) fragments of renal cortico-medullary tissue, including ten glomeruli, of which eight showed advanced ischemic sclerosis, and the rest showed ischemic segmental sclerosis with mesangial alteration. Swelling of the endothelial cells was observed in some capillary loops and some capillaries displayed thickened walls. One glomerulus showed lesions with mesangiolysis. There was marked tubular atrophy with the presence of hyaline casts and interstitial fibrosis, accompanied by abundant lymphocytic infiltrate. Immunofluorescence (IF) revealed albumin positivity in sclerosed glomeruli, while IgA, IgG, IgM, C1q, C3c, and kappa and lambda free light chain, were all positive in tubular casts, but negative in glomeruli (Figure 3). For the electron microscopy, the available material was quantitatively reduced, and a single glomerulus with ischemic sclerotic appearance was examined. One preserved endothelial swelling and cellular interposition at the level of the glomerular basement membrane, with duplication of the membrane.

The detection of TMA in the kidney biopsy raised suspicion for aHUS. Complement analysis and genetic testing were conducted at Semmelweis University, Hungary, but no mutations were detected. 

The identification of thrombotic microangiopathy (TMA) lesions on the renal biopsy rendered the case particularly challenging, raising the issue of differential diagnosis. Although the patient exhibited features suggestive of hemolytic anemia, including the presence of rare schistocytes and mildly decreased haptoglobin levels, the persistently normal platelet count allowed for the exclusion of hemolytic uremic syndrome and suggested that these findings were attributable to the ongoing acute episode. Given the negative results of infectious, immunological, and complement tests, as well as the persistent normal platelet count throughout hospitalization, the possibility that these changes are attributable to malignant hypertension remains under consideration, especially in light of the fact that the patient required continuous intravenous calcium channel blocker to maintain blood pressure within normal limits.

Given the unusual appearance and rapid kidney failure, whole exome sequencing (WES) was conducted. A pathogenic WT1 mutation (c.1298G>A, p.Cys433Tyr) was identified and karyotype analysis indicated 46, XY. At the family’s request, WT1 genetic testing was conducted for both parents and the patient’s sister, returning negative results and establishing a de novo mutation in the patient.

The clinical, pathological, and genetic findings in this patient are consistent with a WT1-related disorder, predominantly aligning with features of Frasier syndrome, while also exhibiting elements characteristic of Denys–Drash syndrome.

**Figure 4:**
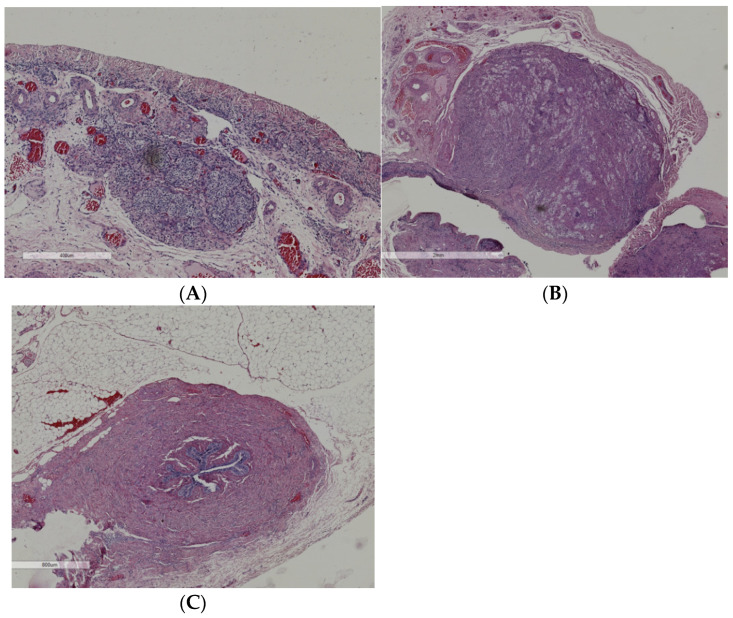
Histopathological analysis of the removed tissue post-gonadectomy: (**A**) right gonad: rudimentary testis predominantly consisting of Sertoli cells; (**B**) left gonad: rudimentary testis containing Sertoli cells; (**C**) left gonad: presence of a deferent duct.

Due to the increased risk of gonadal cancer linked to the WT1 mutation, the patient had laparoscopic bilateral gonadectomy, which revealed rudimentary testicular tissues free of germ cells or malignancy (Figure 4).

One year after diagnosis, the patient continues intermittent hemodialysis and is awaiting a kidney transplant.

## 3. Discussion

The *WT1* gene remains the primary genetic determinant associated with renal pathologies and disorders of sexual development. Comprising 10 exons and spanning approximately 50 kb, *WT1* encodes a nuclear protein characterized by four zinc finger domains, enabling DNA binding and functioning as a transcriptional regulator. WT1-related disorders represent a spectrum of conditions arising from aberrant or absent *WT1* expression [6].

The considerable variability in the age of onset, along with the broad phenotypic spectrum and incomplete penetrance of WT1-related disorders, poses significant challenges in establishing an accurate diagnosis. These factors also hinder a precise estimation of the true incidence of such conditions [1]. In many instances, patients may not present with the complete set of classical clinical features typically associated with specific WT1 syndromes. For example, the absence of the characteristic triad seen in Denys–Drash syndrome (Wilms tumor, nephropathy, and genitourinary anomalies), Frasier syndrome (46,XY gonadal dysgenesis, renal failure, and gonadoblastoma), or the constellation of findings represented by the WAGR acronym can result in a failure to identify individuals affected by WT1 mutations. Consequently, underdiagnosis or misdiagnosis remains a considerable concern in clinical practice [9].

The absence of aniridia, cardiac, and pulmonary malformations, as well as the presence of normal intellectual development, allowed for the exclusion of WAGR syndrome and Meacham syndrome—two WT1-associated disorders typically linked to reduced life expectancy [1,9,10]. The differential diagnosis also considered Nephrotic Syndrome Type 4 but was excluded due to the patient’s phenotype and the confirmed presence of hermaphroditism [11,12].

A notable feature of our case is the presence of a Frasier syndrome phenotype in the context of a Denys–Drash syndrome genotype. Both Denys–Drash syndrome (DDS) and Frasier syndrome (FS) are characterized by male pseudohermaphroditism, progressive glomerulopathy, and an increased risk of genitourinary malignancies. The primary distinction between the two lies in the onset and progression of nephropathy. In DDS, renal disease typically presents within the first year of life and progresses rapidly to end-stage kidney disease (ESKD), often by the age of three. In contrast, FS is usually diagnosed later, with nephropathy manifesting as steroid-resistant nephrotic syndrome that gradually evolves into ESKD within the first two decades of life [13,14,15]. Furthermore, the average age of diagnosis in FS is estimated between 13 and 17 years, often prompted by investigations for primary amenorrhea and delayed puberty, at which point many patients are also diagnosed with ESKD [8,16]. In our case, however, renal function deteriorated so rapidly that there was insufficient time for endocrine manifestations to develop.

The abrupt onset of ESKD at the age of eight, in the absence of prior symptoms such as edema, hypertension, or general health deterioration before the current hospital admission, further supports a diagnosis of Frasier syndrome. Nevertheless, the absence of regular medical evaluations and the presence of concentric left ventricular hypertrophy on echocardiography raise the possibility that the disease may have had an earlier, undetected onset.

The risk of developing Wilms tumor is significantly higher in DDS compared to FS. Most patients with DDS develop Wilms tumor, with a median age at diagnosis around 18 months. Conversely, the risk of gonadal malignancy is notably higher in FS, reaching approximately 60%, compared to about 40% in DDS [15,16,17]. In our patient, the absence of Wilms tumor to date is consistent with a diagnosis of Frasier syndrome, although histological examination of the gonads following gonadectomy did not reveal any evidence of malignancy.

In our case, genetic analysis revealed a heterozygous missense mutation in the *WT1* gene (c.1298G>A, p.Cys433Tyr), located in exon 8, in association with a 46 XY karyotype. FS is frequently associated with mutations in the WT1 gene, particularly those affecting the splice donor site of intron 9 [18]. The genetic mutation identified in our patient is situated in exon 8 and affects amino acid 433. Mutations in this exon have been previously recognized in patients with DDS, and reports of FS resulting from exon 8 mutations in the WT1 gene are infrequent. The majority of reported cases are associated with mutations in intron 9 or exon 9 [19,20]. Additionally, missense mutations in exons 8 and 9 have also been reported in patients with Meacham syndrome [11]. Patients with DDS harboring exon 8 or exon 9 mutations typically present with congenital or early-onset nephropathy, features that were not observed in our patient [21]. In our case, Meacham syndrome was excluded due to the different phenotype. Nevertheless, the genotype identified in our patient is suggestive of DDS, despite the clinical phenotype aligning more closely with FS. However, it is important to note that the genetic testing was limited, as only whole exome sequencing (WES) was performed, not whole genome sequencing (WGS). As a result, this approach may miss mutations located in non-coding regions such as introns.

This example provides novel insights into the genetic underpinnings of WT1 disorders and underscores the necessity of continued study to elucidate the correlation between WT1 mutations and the clinical presentations of this disease. Also, the majority of patients with WT1 disorders possess a de novo pathogenic mutation, leading to an absence of relevant family history. This was verified in our situation, as genetic tests of the family failed to identify the WT1 gene mutation. 

The kidney biopsy indicated advanced global glomerulosclerosis in 8 out of 10 glomeruli and segmental glomerulosclerosis in 2 out of 10 glomeruli, data that support FS [13,18]. In the past, DDS and FS were distinguished by nephropathy patterns: DDS was linked to diffuse mesangial sclerosis, while FS generally exhibited focal and segmental glomerulosclerosis (FSGS). Nevertheless, several cases of DDS associated with FSGS have been documented [15,22]. The results of the kidney biopsy, in our case, indicated thrombotic microangiopathy in 60–70% of glomeruli, suggesting a potential concurrent disease with Frasier syndrome. Nonetheless, immunological assessments, evaluations of ADAMTS13 activity, and genetic investigations for complement system disorders returned negative results. The presence of thrombotic microangiopathy (TMA) lesions may be attributed to malignant hypertension rather than an underlying systemic disease, given the patient’s markedly elevated blood pressure that required continuous intravenous administration of a calcium channel blocker. Nevertheless, the presence of these lesions significantly complicated the diagnostic process.

The late-onset presentation of direct nephropathy progressing to end-stage chronic kidney disease, in the absence of other clinical manifestations until the current episode, the lack of evidence of a renal tumor, and the histopathological findings on renal biopsy are features suggestive of Frasier syndrome. However, genetic analysis supports the diagnosis of Denys–Drash syndrome. The clinical and genetic overlap between these two syndromes lead to ongoing debate regarding whether they represent distinct entities or phenotypic variations within a shared pathological spectrum [6]. Despite the distinct effects these syndromes have on the type of WT1 protein produced, recent studies have shown that mutations typically associated with Frasier syndrome may also result in the nephropathy characteristic of Denys–Drash syndrome, and conversely, WT1 mutations characteristic of Denys–Drash syndrome can give rise to the glomerulosclerosis observed in Frasier syndrome [22,23].

## 4. Conclusions

This case stands out for its complexity, particularly when compared to other reported WT1-related disorders, due to the considerable clinical and genetic overlap between Frasier syndrome (FS) and Denys–Drash syndrome (DDS). The late-onset presentation of direct nephropathy progressing to end-stage renal disease at the age of 8, in the absence of other clinical manifestations until the current episode, the absence of renal tumor, and the histopathological findings on kidney biopsy are indicative of FS. However, genetic testing revealed mutations typically associated with DDS, resulting in a notable phenotype–genotype discordance, with a clinical picture consistent with FS occurring in the context of a DDS genotype.

Another defining feature of this case was the patient’s severe clinical presentation, characterized by malignant hypertension, anasarca, and significant metabolic disturbances, necessitating urgent renal replacement therapy and intensive care. The critical nature of the clinical course underscored the need for a rapid and accurate diagnosis, and emphasized the importance of a multidisciplinary approach involving nephrology, medical genetics, and endocrinology.

A further significant finding was the kidney biopsy result, which demonstrated not only progressive glomerulosclerosis but also features of TMA. Although TMA is not a definitive feature of WT1 disorders, its occurrence prompts compelling inquiries on potential overlapping causes of renal injury, necessitating deeper exploration of suspected secondary pathophysiological processes.

Diagnosing WT1-related disorders remains challenging due to their highly variable clinical presentation. The age of onset can differ significantly among patients. In addition, these syndromes show a wide phenotypic spectrum and incomplete penetrance. These factors complicate efforts to establish a clear and timely diagnosis. To address these challenges, further research is essential. A deeper understanding of the molecular mechanisms involved in WT1-related conditions could improve diagnostic accuracy. Moreover, it may lead to the development of more effective and personalized treatment strategies.

## Figures and Tables

**Table 1:** Preliminary laboratory markers.

Laboratory Markers	Results	References
Hb	7.9 g/dL *	11.5–14.5 g/dL
MCV	78.5 fl	75–89 fl
MCH	27 pg	25–31 pg
Reticulocyte count	0.88 mil/uL	0.03–0.12 mil/uL
Platelet count	280.000/uL	150.000–450.000/uL
Leukocytes	26.810 cel/mm^3^ *	5000–14.500 cel/mm^3^
Neutrophils	24.500 cel/mm^3^ *	1.500–8.000 cel/mm^3^
Haptoglobin	0.15 g/L	0.3–2.0 g/L
LDH	910 U/L *	120–300 U/L
Ferritin	453.8 mg/dL *	7–84 mg/dL
Fibrinogen	661 mg/dL *	140–360 mg/dL
CRP	38.15 mg/L *	0–5 mg/L
Serum urea	281 mg/dL *	<39 mg/dL
Serum creatinine	14.85 mg/dL *	<0.6 mg/dL
Uric acid	8.1 mg/dL *	1.8–5.5 mg/dL
Albumin	3.06 g/dL *	3.8–5.4 g/dL
Amylase	148 U/L *	28–100 U/L
Lipase	80 U/L *	<60 U/L
pH	7.17 *	7.35–7.45
HCO3	16.9 mmol/L *	22–26 mmol/L
pCO2	46.5 mmHg *	37–50 mmHg
Na	132 mmol/L *	135–145 mmol/L
Cl	99.1 mmol/L *	98–113 mmol/L
AG	22.9 mmol/L *	8–18 mmol/L
NT-proBNP	304.486 pg/mL *	<12 pg/mL
Troponin	101 ng/L *	<40 ng/L
D-dimer	6.67 μg/mL *	0–0.5 μg/mL

Hb = Hemoglobin, MCV = Mean corpuscular volume, MCH = Mean corpuscular hemoglobin; CRP = C-reactive protein; HCO3 = bicarbonate; pCO2 = partial pressure of carbon dioxide. NT-proBNP = N-terminal pro B type natriuretic peptide,* = outside the normal range.

## Data Availability

The original contributions presented in this study are included in the article. Further inquiries can be directed to the corresponding author(s).
